# Mechanisms and significance of entosis for tumour growth and progression

**DOI:** 10.1038/s41420-024-01877-9

**Published:** 2024-03-01

**Authors:** Ksenia Аndreevna Gaptulbarova, Irina Alexandrovna Tsydenova, Daria Sergeevna Dolgasheva, Ekaterina Andreevna Kravtsova, Marina Konstantinovna Ibragimova, Sergey Vladimirovich Vtorushin, Nikolai Vasilievich Litviakov

**Affiliations:** 1 Cancer Research Institute “Tomsk National Research Medical Centre of the Russian Academy of Sciences”, Kooperativniy Lane, 5, 634009 Tomsk, Russia; 2 Siberian State Medical University, Moskovsky trakt, 2, 634050 Tomsk, Russia; 3 National Research Tomsk State University, Lenin Avenue 36, 634050 Tomsk, Russia

**Keywords:** Mechanisms of disease, Entosis, Predictive markers

## Abstract

To date, numerous mechanisms have been identified in which one cell engulfs another, resulting in the creation of ‘cell-in-cell’ (CIC) structures, which subsequently cause cell death. One of the mechanisms of formation of these structures is entosis, which is presumably associated with possible carcinogenesis and tumour progression. The peculiarity of the process is that entotic cells themselves actively invade the host cell, and afterwards have several possible variants of fate. Entotic formations are structures where one cell is engulfed by another cell, creating a cell-in-cell structure. The nucleus of the outer cell has a crescent shape, while the inner cell is surrounded by a large entotic vacuole. These characteristics differentiate entosis from cell cannibalism. It’s worth noting that entotic formations are not necessarily harmful and may even be beneficial in some cases. In this article we will consider the mechanism of entosis and variants of entotic cell death, and also put forward hypothesis about possible variants of participation of this process on the formation and progression of cancer. This article also presents our proposed classification of functional forms of entosis.

## Facts

- Entosis is the process by which cells are alive and viable cells are internalised, into neighbouring cells of the same type, with the involvement of adhesion molecules, forming those very cell-in-cell structures.
- The main signalling pathways of entosis are related to the work of proteins of the Rho family of small GTPases, the three proteins most important for entosis are Rho (RhoA, RhoB and RhoC), Rac (Rac1, Rac2 and Rac3) and Cdc42.
- The extent of in vitro models of entosis is currently known. These are deadhesive, mitotic entosis, entosis under glucose starvation and exposure to ultraviolet light.

## Open questions

- We have proposed a functional classification of entotic events for the course of cancer, which still needs to be further reviewed and validated.
- Researchers are still arguing over key distinctions for classifying cell death (emperipolysis, entosis, cannibalism, etc.). This suggests that it is an open question to find the key markers that distinguish one process from another.
- The question of the role of entosis in clinical practice, including prediction of the course of malignant neoplasms, remains open.

## Introduction

To date, there are still many unresolved Issues in the field of light microscopy, one example being the rediscovery of internalisation, the process of incorporating one eukaryotic cell inside another [1]. This term usually describes the static stay of one cell or several cells inside another cell (cell-in-cell; CIC) [1–3]. This particular structure first began to be talked about more than a century ago [4], little was known about its function, process mechanism or consequences at that time.

Subsequently, some terms, such as “bird’s eye cells” or the more common “signet ring cells” [5–7], began to be used to describe similar morphological findings. In which intracellular vacuoles and/or cytoplasmic inclusions are displaced and the nucleus changes its shape. As the number of cases of “signet ring cells” detected increased, the authors began to doubt the appropriateness of this term. And all cases of cell engulfment began to be called cell cannibalism. Later details appeared showing that cannibalism occurs exclusively between identical cell types (homogeneous cannibalism), while in other cases different cell types are involved (heterogeneous cannibalism). Cannibalism can be caused by either an internal cell (internal engulfment) or an external cell (external engulfment) and can either result in cell death or promote cell survival [3]. The mechanisms associated with CIC indicate that not all cases are the same. Different terms and classifications have arisen independently of each other. For example, some authors prefer to call this process cannibalism, entosis, and entosis. Some use the term entosis, others describe it as emperipolysis and so on. The problem of characterisation and identification of CIC structures is still relevant, given these difficulties with their classification and description [1]. Cannibalism, entosis and emperipolysis have differences in their mechanisms of formation, intercellular relationships and intracellular fate. Nevertheless, since there are no established definitions of these terms, many authors have expressed conflicting opinions as to what constitutes a CIC event [1, 8]. CIC events can be readily identified through haematoxylin-eosin staining and are frequently observed in cancerous tumours [9], including lung cancer [10]., breast cancer, melanoma, and even benign tumours [1].

At first, CIC structures were regarded as a mere biological curiosity, only familiar to a small group of specialists. Nonetheless, growing interest in CICs implies that they could be a novel essential element in regulating the survival and death of cells. Research publications indicate that the formation of CICs might correlate with the progression and degradation of cancer outcomes [11–13]. Furthermore, research indicates that regulating and targeting this process may serve as a potential therapeutic approach to impact cancer patients’ prognosis. This paper aims to explore various facets of CIC, including its significance and role in tumour progression and evolution. Biological term abbreviations will be explained upon their first use, and consistent citation and formatting features will be used throughout.

### Classification of cell death, place and classification of entosis

There are considerable difficulties in developing a single classification of all cell death types. The world uses an approach related to structural differences in the realisation of different death scenarios. Cell death is marked by visible morphological changes and mechanisms for disposing of dead cells and their components. A widely utilised classification for cell death encompasses three types [14]: apoptosis is the first type, autophagy is the second, and cell necrosis is the third. Despite a number of limitations and caveats, this classification, enjoys considerable popularity. It is based on morphologic features. The basic principles of this classification relate to the specific signal transduction pathways that control the initiation, execution and propagation of cell death. It is also related to the pathophysiologic significance of the main types of regulated cell death. In their review, Galluzzi et al. consider the presence of both regulated cell death (RCD) and accidental cell death (ACD). On the one hand, regulated cell death can be classified into two subtypes [14]: programmed cell death and unprogrammed cell death. Entosis is classified as “A type of RCD that originates from actomyosin-dependent cell-in-cell internalisation (entosis) and is executed by lysosomes”.

The review by Deev et al. also presents a morphological classification. However, the imprecision of criteria and the difficulty of differentiating the variety of death types by morphological criteria make its application very limited [15]. Entosis in this classification is referred to the types of pathological histogenesis cell death, motivated by the fact that it occurs most often, in pathological tissues, such as tumour.

In a review paper, Yan et al. also presented their generalised qualification of cell death processes. Firstly, they divided cell death processes into 2 groups: programmed or unprogrammed cell death based on their dependence on signals. Programmed cell death (PCD) is caused by regulated intracellular signal transduction pathways, while unprogrammed cell death, which results from unexpected cellular damage [16], is referred to as accidental cell death. Based on morphological characteristics and molecular mechanisms, programmed cell death can be subcategorised into apoptotic cell death and non-apoptotic cell death. Apoptosis occurs in a caspase-dependent manner and is responsible for maintaining cell membrane integrity. In contrast, non-apoptotic cell death is mainly characterised by membrane rupture and is independent of caspase activity. Entosis, in this classification, denotes vacuole-dependent, non-apoptotic programmed cell death [16]. As can be seen, each of these classifications distinguishes entosis of interest as a separate and independent process, which indicates the relevance of its study.

Entosis is described in the literature as a relatively new way of formation of cell-in-cell structure that bears a resemblance to typical CIC structures. Compared to cannibalism and emperipolysis, entosis is a recently developed concept in which one living cell invades another cell, resulting in the destruction of the internalised cell [9]. Morphologically, the outer cell nucleus is shifted towards the periphery and has a semilunar shape. In our opinion, the following distinctive features should be introduced to clearly distinguish these processes (Table 1).

**Table 1:** Main differences between cannibalism, emperipolysis and entosis.

Cellular cannibalism	Emperipolysis	Entosis
The host cell actively absorbs another cell	The internalised cell itself penetrates the host cell	The internalised cell itself penetrates the host cell
Absorption of heterogeneous and homogeneous cell types (neutrophils, lymphocytes and erythrocytes)	Absorption of heterogeneous (lymphocytes and other blood cells)	Absorption of homogeneous cell types only
Absorption of both dead and living cell	Absorption of living cells only	Absorption of living cells only
*Internalised cell:* - digested by the host cell	*Internalised cell:* - kills the host cell - is destroyed by the host cell (lysosomal, apoptotic death)	*Internalised cell:* - lysosomal death - apoptotic death - division inside the host cell, - stays inside host cell unchanged for more than 24 h and leaves host cell

According to this classification, entosis and emperipolysis differ from cannibalism in that in these processes the cell itself invades the host cell. Rather than being actively engulfed as in cannibalism. Emperipolysis differs from entosis in that in emperipolysis it occurs between heterotypic cells, whereas in entosis it occurs only between homotypic cells. In addition, in emperipolysis, the internalised cell can kill the host cell.

In addition to these distinctions, we will argue that the process now called entosis is not a form of cell death. Rather, it is a form of cell behaviour and consists of several different cell-cell interactions involved in entosis. The functional significance of each of them determines its essence and should have its own name. Our classification is based on variants of cell-cell interaction or internalised cell fate (Table 1). The variants of cell-cell interactions, which define the essence of the process, define the gap between infiltration and the outcome of cell-cell interaction. Since it is known that the death of an internalised cell, although frequent, is not always inevitable [17]. Thus, an internalised cell may be viable for many hours and can escape. As reported by Overholtzer et al. 12–18% of internalised cells were eventually released in MCF7 and MCF10A cell cultures, respectively. These cells appear normal after release and are able to divide. Moreover, a small percentage of internalised cells can divide within the host cell (9% and 0.8% for MCF7 and MCF10A respectively) [9]. The form of internalised cell death within the host cell may also vary. They may undergo lysosomal autophagy-like death with cytoplasm vacuolisation or apoptotic death. Entotic cell death is usually the most common fate [17]. Garanina et al. described five stages of entosis in the adherent cell monolayer after formation of cell-in-cell structure. This classification is based on the study of morphological changes during entosis [18]:

1. In the first stage, the inner cell is round, with a round and unchanged nucleus. The size of the inner cell is similar to that before internalisation, and its plasma membrane is close to an entotic vacuole with the presence of intercellular connections.
2. In the second stage, the cell shrinks and the vacuole enlarges.
3. In the third stage, the internalised cell becomes irregularly shaped and exhibits chromatin condensation.
4. In the fourth stage, the cell and its nucleus become deformed and the nuclei disappear.
5. In the fifth and final stage, only the vacuolised cytoplasm and condensed chromatin remain of the inner cell

It has been shown that in the final step, if the internalised cell has not left the host cell, it is destroyed by lysosomal death or, more correctly, by selective autophagy, using appropriate proteins [8, 19, 20].

**Fig. 1:**
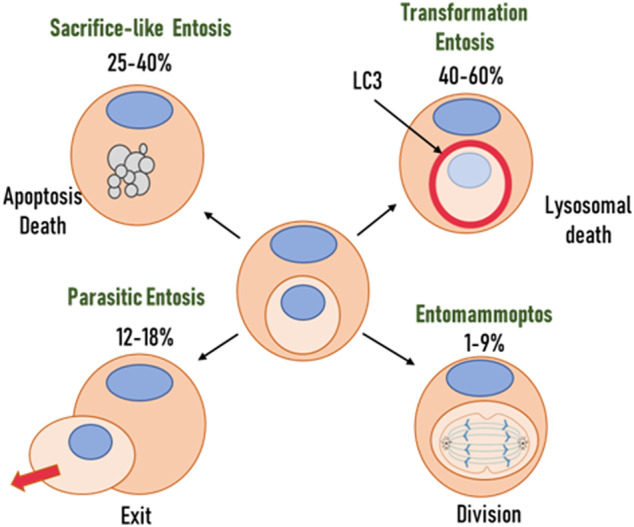
Functional classification of entosis forms.

If we base the classification of entosis on the cell-cell interaction variants, the following hypothetical considerations can be made (Fig. 1):

1. The cell penetrated into another cell and left it after some time. It can be assumed that in this way the internalised cell survived unfavourable conditions for itself. At times when it was vulnerable (for example, due to the action of xenobiotics). The internalised cell used the external cell as protection and possibly food. This type of cell-cell interaction can be compared to obligate parasitism, and we would call it parasitic entosis. Another suggestion is that privileged tumour stem cells may use such a defense mechanism.
2. A cell has infiltrated another cell and started dividing inside it. In fact, the outside cell acts as a cradle for the inside cell and we would call this type of interaction entomammoptosis. Here, we can assume that the internalised cell has de-differentiated and become a stem cell.
3. A case when a cell has invaded another cell and died as a result of apoptosis. In doing so, the entire cell, including the nucleus, has degenerated. The remnants of the inner cell remain inside the host cell for possible nourishment. It is as if the cell sacrificed itself for the survival of the engulfing cell. We have called this variant sacrificial entosis. In this case, we can assume that the engulfing cell is a TSC and the neighbouring cell sacrifices itself for its survival.
4. A case where a cell penetrates another cell and dies by lysosomal death or selective autophagy. This too is clearly a sacrifice, but there is evidence that in addition to feeding, cell nuclei fusion may also occur in this case. And the engulfing cell may change at the genetic level, becoming poly- or aneuploid and acquiring greater resistance to external conditions. This variant of entosis we would call transformational entosis. Again, the external cell is most likely a stem cell.

This still needs to be studied; no data are available in the literature on the subpopulation of cells participating in entosis.

As we suggest, depending on the type of entosis, its role in tumour development and progression will differ, which we will discuss below.

### Entosis. The general mechanism of entosis and its main elements

The integrity of all epithelial tissues depends on the absence of damage to adhesive junctions. This is conditioned by the recruitment of cadherins lining the intracellular space and cytoplasmic catenins that attach their cytosolic tails to actin filaments and further depend on actin-myosin contraction regulated by Rho GTPases [21]. During entosis, Rho activation occurs in a cell that has lost contact with the basal membrane, after which the cell forms adhesive junctions with a neighbouring cell and actively invades it, forming a cell-in-cell structure [9, 13]. In other words, rather than providing “eat me” signals, entotic cells themselves provide the key driving force through actin polymerisation and myosin contraction to facilitate the ingestion process itself [11].

**Fig. 2:**
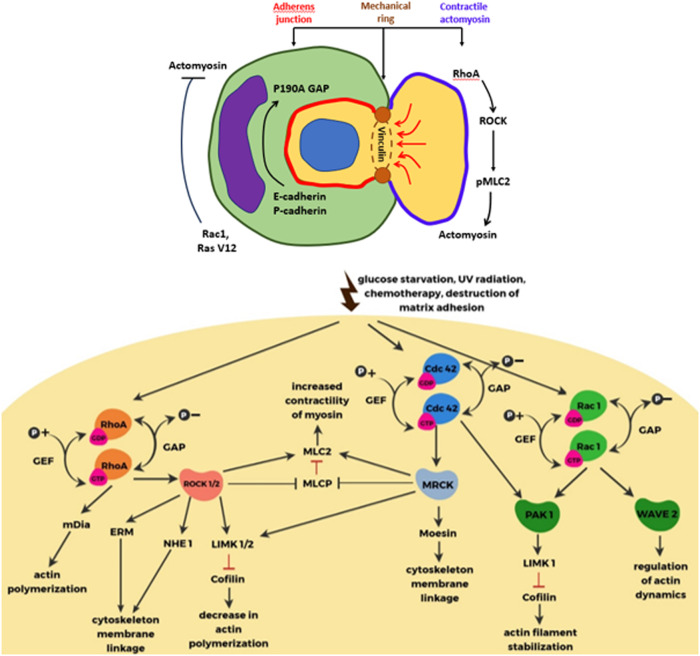
The main known molecules regulating entosis. Note: The central part is a schematic representation of the intercellular interaction during entosis. On the left, the inner cell rounds off after losing contact with the substrate. On the right, the outer cell wraps around the inner cell and has a sickle-shaped nucleus. The two cells interact by forming adhesion contacts mediated by E-cadherin, β-catenin and calcium ions. The AMPK protein kinase is active in the outer cell and inactive in the inner cell. On the left is a list of molecules that are active in the inner cell. The arrow indicates the factors that cause cancer cell entosis [13].

The family Rho of small GTPases includes many proteins, but three proteins are most important for entosis: Rho (RhoA, RhoB, and RhoC), Rac (Rac1, Rac2, and Rac3), and Cdc42 [21, 22]. These proteins are involved in cell adhesion and migration. Rho GTPase is thought to be active when bound to GTP and inactive when bound to GDP. It can cycle between these two states, and this process is regulated by two opposing families of proteins: guanine nucleotide exchange factors (GEFs) and GTPase activating proteins (GAPs). They are required to separate tightly bound GDP from GTPase and to stimulate the hydrolysis of GTP to form GDP (Fig. 2) [21]. Rho-GTP activates its main effector, Rho-associated protein kinase (ROCK). This leads to phosphorylation of myosin light chain 2 (MLC2) and repression of MLC2 phosphatase. Which promotes the interaction of actin with myosin II and increases contractility, actin fibre formation and focal adhesion. Phosphorylation of MLC2 at Ser19 and Thr18 affects the ATPase activity of myosin heavy chain (MHC), which moves along actin filaments to generate contractile force [23]. The intercellular interaction attracts Rho-GAPs (RhoGTPase-activating proteins that promote the GDP-bound inactive conformation of Rho), locally inhibiting Rho activity and creating a gradient of active Rho in the internalising cell. As a consequence, the concentration of active Rho becomes higher at the opposite end of the cell from the intercellular contact. This creates mechanical tension leading to intracellular invasion. Purvanov et al. in their study described that the invading cell demonstrates swelling of the plasma membrane with subsequent actin assembly in the posterior part, forming a uropod-like, actin-rich structure that promotes invasion [24, 25]. Interestingly, the difference in membrane stiffness between the internalised cell and the host cell determines whether CIC formation will occur. If the difference in stiffness between cells is sufficient to overcome the energy barrier, CIC formation becomes possible, and cells with higher RhoA activity could penetrate into neighbouring cells [25, 26]. Все названия в этой статье [26].

Adhesion junctions between cells play an important role in the process of entosis. They also demonstrate asymmetric distribution within the cells involved in the formation of CIC structures [26]. Tumour cells lacking expression of adhesive junction components (E-cadherin, P-cadherin, or α-catenin) did not form CICs. Restoration of the expression of these molecules in the corresponding cells, again induces the formation of entotic CICs [26, 27].The adhesive compound was also found to possess subcellular localisation. It promotes the establishment of polarised actin and myosin distribution in the posterior periphery by recruiting RhoGAP p190A. RhoGAP p190A is a potent inhibitor of RhoA activity by converting RhoA-GTP to RhoA-GDP [9, 11, 26–28].

But the question remains as to what lies at the interface of the adhesive transition and contractile actomyosin. Wang et al. showed that ectopic expression of E-cadherin in MDA-MB-231 cells deficient in E-cadherin expression can effectively induce entotic CIC formation [27, 29]. Exogenous GFP-labelled E-cadherin not only labelled the cell-cell contact region, but was also specifically enriched in two cortical foci between the inner and outer parts of the inner cell. Projection of the foci onto the composite 3D image allowed us to obtain a ring-like entrance for cell internalisation. The study of CIC structures at different stages showed that the ring-like structure is present throughout the entire process of CIC formation from the beginning of internalisation to complete closure [29].

To investigate the molecular basis underlying this ring-like structure, Wang et al. tested the presence of adhesive and cytoskeletal molecules. At the late stage of entosis, typical structures could be easily detected for most molecules, including a-catenin (a-CTN), b-catenin (b-CTN), g-catenin (g-CTN), a-tubulin, and F-actin. The most curious component turned out to be vinculin, which, along with g-CTN and F-actin, formed the ring-like structure more often than others. It also showed the highest enrichment in the ring-like structure. Vinculin itself is a force-sensitive protein that stabilizes E-cadherin-based cell-cell adhesion. And enhances the interaction between a-CTN and F-actin [26, 29, 30]. When examined by structured illumination microscopy (SIM), it became clear that the transmembrane protein E-cadherin was (usually) spatially alternating with cytosolic vinculin. Although in places they were partially co-localised [29].

Thus, we have considered the main components of the mechanism of invasion of one cell into another. It can be seen that the main driving force is represented by activation of the Rho-ROCK pathway and contractility of actomyosin in the invasive cell. As well as differences in deformability of the host cell and the internalised cell. It is important to note that the inner cell actively participates in the initiation of the whole process and its realisation.

### Basic entosis models

**Fig. 3:**
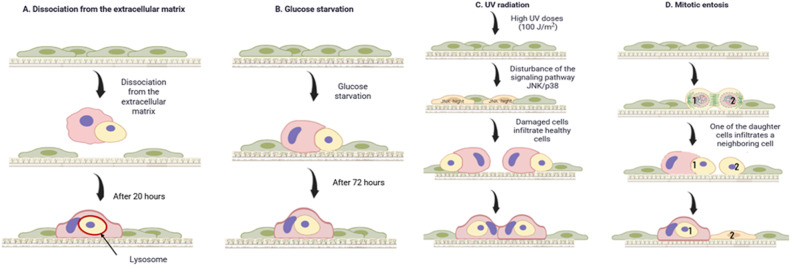
Known models of entosis in vitro to date.

Several in vitro models of entosis are currently known and are shown in Fig. 3. These are de-adhesion, mitotic entosis, glucose starvation entosis and ultraviolet exposure.

De-adhesion is considered a basic type of entosis to maintain the integrity of tumour tissue. It is well known that cells undergo apoptosis if they are not attached to the extracellular matrix (ECM) or if they attach to inappropriate sites (anoikis) [31]. In tumour tissue, these processes are usually impaired or suppressed. It has been shown that entosis compensates for defects in apoptosis in individual cell populations [9]. The breakdown of matrix adhesion is probably caused by entosis. Since this phenomenon is mainly observed in suspended samples such as liquid exudates, urine and bile [32, 33]. Overholtzer et al. first identified entosis in mammary epithelial cells detached from the matrix in vitro [1, 9]. After separation from the matrix, the establishment of adhesion between cells that participate in entosis is an important step towards the formation of cell-in-cell structures [34]. E-cadherin and P-cadherin are key components of cell adhesion. Intercellular structures cannot be formed in tumour cells when insufficient amounts of these elements are present. However, they can be formed when E- or P-cadherin is re-expressed [1]. Another study showed that alpha-catenin is also linked to cell formation in cells [35]. After adhesion between cells, entosis is triggered by an imbalance of actin-myosin contractions between neighbouring cells [8]. This process is mediated by the small GTPase RhoA and its effector kinase rho-kinase (ROCK I/II) [9]. When they are inhibited, the formation of intercellular structures is blocked [32, 34]. Subsequently, cells trapped by entosis die, being destroyed by a non-apoptotic lysosomal pathway [1].

Mitotic entosis has only recently been discovered and it can occur when cells attach to the matrix [18, 36]. Under conditions of attachment to the matrix and preservation of tissue integrity, mitosis is an inducer of entosis. In a study by Siquara da Rocha et al., entosis was shown to occur in human bronchial epithelial cells in adherent culture. By following these cells, they found that the engulfed cells undergo mitosis or undergo mitosis just prior to entosis [1].This process is regulated by the protein cdc42.It plays a role in the attachment of cells to each other and to the extracellular matrix. It has been shown that cdc42 enhances mitotic degradation of adhesion and accounts for its rounding. This may be because cdc42 inhibits mitotic activation of RhoA in polarised epithelial cells. And its loss leads to hyperactivation of RhoA during metaphase [32, 34], which leads to entosis. Inhibition of mitosis led to a decrease in the appearance of cell-to-cell formation among cells attached to the matrix. This suggests that mitosis may induce entosis in adherent cell populations. Thus, matrix detachment and mitosis are factors that induce entosis. In the work of Durgan et al. several variants of cell internalisation into the host cell were observed: when one or both daughter cells invaded one or different hosts, or one daughter cell invaded another and both entered the attached neighbour [37]. In the same work, the fate of 54 inner cells of the MCF7 cell line was analysed after 20 h. Of these, 63% underwent apoptotic or non-apoptotic cell death, 22.2% left the host cell, and 14.8 remained intracellularly unchanged. In host cells, subsequent division processes were most frequently disrupted [37].

Another option for modulating entosis is achieved by deprivation of nutrients, particularly glucose. It has been shown that removal of glucose from the culture medium leads to a high level of entosis. Repeated addition of glucose to the medium inhibits this process [32, 38]. For example, Hamann et al. cultured MCF-7 human breast tumour cells. The culture showed a high level of entosis under various nutrient-deficient conditions. After 72 h in medium without glucose and amino acids, neighbouring cells engulfed each other at a high rate. On average, cells with two engulfed neighbours were observed. More complex cell structures with three or more cells were also observed. Interestingly, removal of glucose from the medium, as opposed to starvation in the absence of other nutrients, was sufficient to induce high levels of cellular uptake. And repeated addition of D-glucose to the medium without glucose prevented this process [38], which is associated with the Warburg effect. Thus, the study found that after 10 h of cultivation, 90% of entotic cells in glucose-free medium were killed by lysosomal digestion (as this process was accompanied by lipidation of LC3 in entotic vacuoles). Glucose starvation was also found to stimulate the development of two mechanistically distinct cell populations. One demonstrates relatively greater deformability (low elastic modulus), the other less deformability (high elastic modulus) [38].

A model showing in vitro that entosis can be induced by UV radiation was demonstrated in Chen et al. MCF7 human breast cancer cells, which are capable of inducing entosis [9, 38], were exposed to increasing doses of UV radiation. UV irradiation induced dose-dependent entosis, as more than 35% of cells were involved in entosis after treatment with a high dose of UV irradiation (100 J/m2). Entotic structures were identified by the localisation of the intercellular adhesion protein E-cadherin at the interface between invaded cells and outside cells. Knockout of the *Cdh1* gene encoding E-cadherin also inhibited UV-induced entosis. It could be restored by re-expression of E-cadherin [19].

It has been shown that entosis can occur in normal tissues in some cases, including during embryo implantation [22, 39] and the development of *Caenorhabditis elegans* [40]. However, this phenomenon is most often observed in tumour tissues, where it contributes to the more aggressive nature of the disease. As it stimulates competition for survival between individual cells [19, 41].

It is important to note that the unbalanced strength between two interacting cells, which exceeds the strength of the cellular matrix, is the most fundamental and critical reason for the formation of entotic structures. Deadhesion is rather an extreme situation in which the strength of the cell matrix is equal to zero.

Entosis requires the formation of adhesive junctions in the absence of integrin signals and the forceful invasion of one cell into another [1, 33]. In addition, this process requires the presence of the Rho-ROCK signalling pathway in the internalised cell and muscle contraction in the host cell. It is less clear which and how much upstream signalling can induce this process. As well as what markers of entosis currently exist for its identification [9, 42, 43].

### Entosis inducers and inhibitors

In the study by Solovieva et al. a 6-h incubation of MCF-7 cells with DDC+ B12b (diethyldithiocarbamate combined with vitamin B12b) was performed. After the remaining live cells were washed from DDC+B12b and the response of cells in control was observed after 6, 24 and 48 h. Most cells had a cell-to-cell morphology characteristic of entosis. Triple or quadruple entosis structures were often present. The outer cells had sickle-shaped nuclei, and some of the engulfed cells and host cells had vacuolized cytoplasm [44]^.^

One study demonstrated that nintedanib can promote entosis in prostate cancer treatment by modulating the *CDC42* pathway (hence mitotic entosis), leading to drug resistance [45]. Entosis may provide a safe environment in which the tumour cell can avoid exposure to chemotherapeutic agents [46]. On the other hand, in one particular case of breast carcinoma, extensive lymphocyte emperipolysis was observed. This tumour was virtually destroyed by chemotherapy before surgery, suggesting that emperipolysis could enhance the cytotoxic effect of chemotherapy, possibly through the release of cytokines [47]. Thus, the cell-in-cell phenomenon, depending on the species, can regulate tumour sensitivity to chemotherapeutic drugs in different ways, both by increasing tumour resistance (entosis) [45, 48] and reducing it (emperipolysis) [47]. The development of therapeutic strategies based on the CIC phenomenon should take this into account.

Many known tumour markers, oncogenes and suppressor genes are associated with the process of entosis. The tumour suppressor *CDKN2A*, involved in entosis, has been described. It was described in the work of Mackay et al. Inactivation of *CDKN2A* gene promotes entosis, while *CDKN2A* expression on the contrary is associated with CIC formation in breast cancer [49]. Oncogenes are also involved in entosis, such that overexpression of *KRAS V12* or *c-Myc* has been shown to promote the formation of CIC structures [50]. Importantly, the expression of *c-Myc, KRASV12* and mutant *p53*, as well as the loss of *CDKN2A*, are characteristics of the outer cells of the CIC structure [25, 49–51]. This shows that entosis can influence the positive selection of “winning” tumour cells. Those that have acquired mutations in *c-Myc, KRAS, CDKN2A* or *p53* (hence more malignant). Which leads to the heterogeneity seen in many tumours. In this regard, entosis is now considered a significant factor in the development of intratumoural heterogeneity.

In earlier work, Basbous et al. studied the role of *Rnd3* expression in HCC progression. They found that silencing of *Rnd3* leads to CIC and resembles entosis in terms of detection criteria. In a cellular experiment, knockdown of *Rnd3* led to a significant increase in cell-in-cell events in Hep3B and Huh7 cells. Neighbouring cells engulfed each other (about 10% of adherent cells contained engulfed neighbours). Thus, CIC events, under modelled loss of *Rnd3*, share common features with entosis. Which is observed in nutrient deficiency or lack of attachment to the matrix [52].

Interesting data were presented in Wang et al. Cells overexpressing the multidrug resistance protein P-gp showed a 2.8-fold decrease in the rate of entosis compared to the control. Whereas knockdown of 4,1 N (a product of the *EPB41L1* gene scaffold protein) in the SKOV3 cell line resulted in a 7.9-fold increase. Since entosis depends on Rho-ROCK activity we showed that loss of 4,1 N expression increases the levels of RhoA and phosphorylated myosin light chain 2. This leads to active entosis. And treatment with ROCK inhibitor Y27632 had a dose-dependent inhibitory effect on entosis in cells without 4,1 N expression [53].

In studying the molecular mechanisms underlying the formation of homotypic CIC structures (homoCIC), Wang et al. performed an experiment. In which they knocked out *PCDH7* expression in MDA-MB-436 cells. As a result, depletion of *PCDH7* significantly increased homoCIC formation. To further confirm the effect of *PCDH7* on homoCIC formation, all four *PCDH7* isoforms were overexpressed in MCF7 cells. *PCDH7* is expressed at the lowest level in this cell line. Expression of all isoforms effectively suppressed homoCIC formation in MCF7 cells. These data indicate that *PCDH7* is a negative regulator of homoCIC formation [54]. These findings are supported by a recent study by Liu et al. Their aim was to investigate the function and mechanism of action of protocadherin 7 (*PCDH7*) in colorectal cancer. *PCDH7* overexpression promoted proliferation and invasion, altered autophagy, and induced ferroptosis and homoCIC [55].

### Entosis markers

In addition to morphological signs of entosis, reliable molecular markers of this event should be sought. A single molecular marker of entosis does not exist to date. There are several different approaches to the identification of entosis. First of all, the LC3 protein, a component of autophagosomes and a marker of autophagy. It can be used as a marker to distinguish between apoptotic and entotic internalised cell death. In entotic internalised cell death, lipidation of LC3 on the outer membrane of these cells is observed, which usually occurs prior to internalised cell death. During autophagy, lipidisation of LC3 occurs within cells. During entosis, lipidisation is accompanied by the fusion of lysosomes that acidify entotic vacuoles. This leads to non-apoptotic cell death. Since in some cases the autophagy pathway may contribute to cell death, Florey et al. investigated autophagy during entosis death. They used frame-by-frame imaging of the autophagosome marker GFP-LC3. They found rapid and temporary incorporation of GFP-LC3 from host cells into the membranes of entotic vacuoles [22, 42].

Similarly, delayed visualisation of fluorescently labelled lysosomal proteins such as LAMP1 (lysosome associated membrane protein, a marker of lysosomes) or β-catenin expressed in host cells. They can be used as an indicator of lysosome fusion with the entotic vacuole. Florey et al. showed that LAMP1-GFP is incorporated into the entotic vacuole approximately 30 min after LC3 before internalised cell death. By expressing mCherry-labelled cathepsin, Florey et al. confirmed that lysosomal enzymes from the host cell are deposited in the entotic vacuole prior to internalised cell death. The conversion of entotic vacuoles into lysosomal compartments may indicate that entotic cells are killed by their hosts. And the above-mentioned proteins can be used as markers of this process [42].

Monitoring the acidification of vacuoles containing inner cells can be used as an alternative approach to mark entotic cell death. Acidification can be monitored by adding fluorescent LysoTracker dyes (Thermo Fisher) to the medium during interval imaging analysis, acidification of the entotic vacuole leads to increased and diffuse LysoTracker staining inside the internalised cells [9]. Another marker of entosis may be AMPK, which was used in their study by Hamann et al. To determine whether AMPK activation occurs in winning or losing cells, Hamann et al. used a Foerster resonance energy transfer (FRET) sensor to monitor the temporal dynamics of AMPK. FRET-based fluorescence (indicating increased AMPK activity) in internalised MCF-7 cells induced by glucose starvation increased shortly before infiltration [38]. Determination of entosis histologically is still difficult. Only CICs can be identified morphologically more or less reliably. Clear morphological identification of the different types of CIC on sections is often very difficult. This limits clinical studies of the relevance of entosis to tumour growth and progression. In addition, while markers indicating the onset of entotic internalised cell death are available for experimental work, no such predictive markers are available for other forms (exit - parasitic entosis and entomammoptosis). And these forms of behaviour can be observed only with frame-by-frame imaging.

### The role of entosis in carcinogenesis

Literature data on the possible involvement of entosis directly in carcinogenesis are scarce. It is known that gastric cancer (GC) occurs as a result of the gradual development of atrophic gastritis, intestinal metaplasia (CM (IM)), low grade dysplasia (LGD) and high grade dysplasia (HGD) [56]. In their study, Kim et al. report the effect of high expression of *CDC20* on the incidence of cell-in-cell structures. Interestingly, almost all external host cells overexpressed *CDC20* (cell division cycle protein 20) during CIC formation. Meanwhile, the internalised tumour cells did not express *CDC20* at all. It is also noted that “winning” cells engulfed not only neighbouring homotypic tumour cells, but also apoptosis-prone cells, heterotypic immune cells (histiocytes, lymphocytes and neutrophils). The frequency of CIC formation was shown to increase dramatically with high grade dysplasia (27/50; 54.0%). CIC structures were most frequent in early gastric cancer (205/345; 59.4%) and decreased in late gastric cancer (99/313; 31.6%) [57]. This study, although indicating a high level of entotic activity in precancerous gastric cancer and associated with the process of transition to a malignant form and *CDC20*, according to the authors, can be used as an immunohistochemical marker for the isolation of CIC structures.

### Role in tumour progression

The significance of cell-in-cell structures for tumour progression is rather twofold. In the first case, such structures may slow tumour progression by promoting the death of internalised cells. For example, a recent study showed that cancer cells engulfing mesenchymal cells (stromal/stem cells) are associated with tumour dormancy. And inhibit tumourigenicity rather than cancer cell proliferation [58]. On the other hand, these entities may promote tumour progression by digesting unwanted internalised cells, thereby screening out tumour cells with low malignancy through cell competition [32], cell-in-cell structures are most common in more aggressive cancers. This suggests that the cell-in-cell phenomenon may generally benefit the tumour and contribute to tumour progression [34]. If we consider the cell-in-cell phenomenon as a form of competition between tumour cells, it can be thought that it may contribute to tumour progression in two ways: 1) “winning” cells have a high level of cytokinesis failure. This leads to aneuploidy and increases their malignant potential; 2) the “winning” cells receive nutrients from the cells they have engulfed. This allows them to survive and proliferate in the absence of nutrients [59]. As discussed above, the cell-in-cell phenomenon leads to increased CIN (chromosomal instability) due to disruption of mitosis. In addition, chromosomal components of engulfed cells can be easily detected in host cells. This suggests that chromosome exchange occurs between engulfed and host cells. Inflammation-induced tumour progression may be due in part to inflammatory factors such as IL-6 and IL-8. As they favour the formation of cell-in-cell structures and hence CIN [32, 60].

A recent study has shown that *p53* mutations, which tend to increase tumour malignancy and promote recurrence, are associated with an increased incidence of cell-in-cell structures in patients with lung adenocarcinoma [51]. A recent study has shown that *p53* mutations are associated with an increased frequency of intrinsic structures in patients with lung adenocarcinoma. Mackay et al. used A431 cells (p53 273H) transfected with eGFP, mCherry plasmids or CRISPR constructs to knockout *p53*. In vitro, mixed populations of p53 mutant and p53 null cells exhibited a higher frequency of CIC than p53 null or p53 mutant cells individually. A431 R273H-mutant *p53* cells were more frequently observed as outer cells in definitive CICs. In addition, genomic instability was increased in lung tumours with a higher prevalence of CIC structures. These results suggest that CICs may be responsible for intratumoural heterogeneity [46, 51].

A clinical case demonstrated by Ruan et al. The paper presents a breast cancer with a high degree of heterogeneity, high proliferative activity of tumour cells and high frequency of CIC structures, which could lead to high tumour resistance to several chemotherapy regimens and poor outcome [61].

**Fig. 4:**
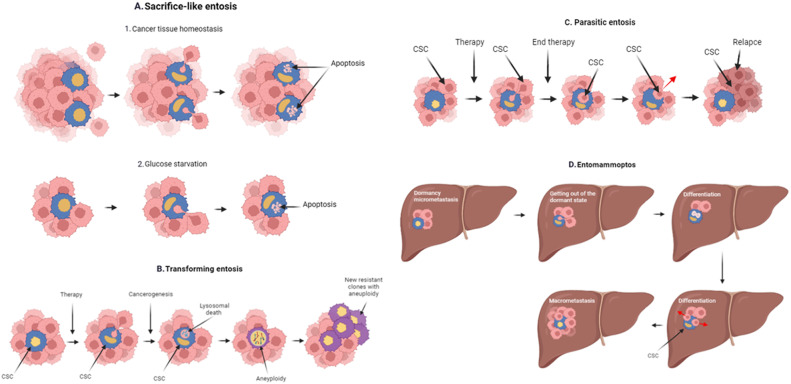
The role of entosis in tumour growth and progression. **A** Sacrifice-like entosis can be used to nourish tumour stem cells during periods of nutrient deficiency or exposure to therapy; **B** Transforming entosis may play a role in tumour progression. In particular, fusion can occur and aneuploidy can form. This leads to the formation of new tumour clones; **C** Parasitic entosis may contribute to the survival of tumour cells during the period of therapy. And once they form a recurrent clone; **D** Entomammoptosis may play a significant role in the dedifferentiation of micrometastasis cells. The formation of CSCs (cancer stem cell). And subsequently transform micrometastases into macrometastases.

Initial suggestions have been made about the role of entosis in tumour growth and development. It depends on the fate of entotic cells and the species (or model of entosis). On the one hand entosis can both inhibit tumour progression by killing tumour cells detached from the matrix. On the other hand it can promote progression by replenishing nutrients from internalised cells or by altering cell ploidy (Fig. 4) [32, 62].

We present our presumptive judgement in this regard. The role of entosis in tumour growth and progression depends on the type of tumour according to the classification we have proposed above. Entosis may play different roles at different stages of tumour development. Sacrifice-like entosis (inner cell death as a result of apoptosis), serves for survival in conditions of temporary lack of nutrition. It is involved in maintaining the tumour stem cell population (presumably external cells). On the other hand, external cells may act as cells to maintain tissue homoeostasis. In this way, cells detached from the matrix are destroyed under conditions of de-adhesive entosis. In this case, entosis reduces the number of tumour cells and is favourable (Fig. 4A).

Transforming entosis (inner cell death by a type of lysosomal death) contributes to the progression of tumour evolution. This type of entosis can lead to aneuploidy by blocking cytokinesis, a steric interference with the normal dynamics of cell division [62]. Or, as we hypothesise, by directly joining the genetic material of the inner and outer cells (Fig. 4B).

It was shown that cells with knockout *p53* often died after engulfment by other cells. In contrast, cells with mutant *p53* usually survived and often continued to undergo abnormal mitotic events. And led to multinucleated and tripolar mitotic events. The mutant form of *p53* solved the problem of abnormal mitotic events, allowed to pass through mitosis checkpoints. Otherwise, the error would have led to apoptotic death of the abnormal cells. And this would have led to chromosomal changes [2, 63].

Transforming entosis is common in pre-tumour malignancies and in the formation of resistant clones during chemoinduced tumour evolution. This is supported by a study by Durgan et al. who showed that taxanes can significantly enhance mitotic entosis and the formation of aneuploid tumour cells [37].

Numerous studies reviewed by Ibragimova et al. show that 10 to 50 per cent of tumours acquire new mutations (very rapidly, even after a single course) under the influence of chemotherapy and targeted therapy. Including amplifications of large chromosomal regions that lead to the formation of more malignant phenotypes [64]. We have also previously shown that the presence of two or more amplifications of stemness genes in the tumour of breast cancer patients (which can also form during neoadjuvant chemotherapy) is associated with a high rate of distant metastasis [65, 66]. We looked at the CNA genetic landscape of the tumours of the examined patients and found that deletion of the *CDC42* gene locus (1p36. 12), inhibition of which induces mitotic entosis [37] was associated with a high rate of stemness gene amplification. In the delCDC42 group of patients, the frequency of tumours with 2 or more amplifications of the stemness gene (a very poor prognostic factor) was 78% (28/36), versus 58% (63/108) in the normal or amplified *CDC42* group (*p* = 0.045).

According to the presented logic, transforming entosis leads to tumour evolution. And it should increase in the process of malignisation and tumour treatment, but not in the process of growth of a formed tumour. This agrees well with the work of Kim et al. who showed that entosis increases as gastric precancer maligns [57]. After the formation of malignant gastric cancer, there is a change in the types of entosis from transforming to sacrificial entosis. Its high frequency indicates destructive processes in the tumour and leads to increased patient survival. Assuming that such a change of entosis types does not occur in tumours of other localisations, its unfavourable role noted by many researchers can be easily explained [4, 6, 67, 68].

We hypothesise that a parasitic type of entosis serves for the survival of tumour stem cells during drug exposure. In this case, the internal cells benefit. This type of entosis should be associated with tumour recurrence. Confirmation of this has yet to be found or refuted (Fig. 4C).

The last type of entosis in our proposed classification is entomammoptosis.In it, the inner tumour cell divides. We believe that a differentiated tumour cell enters the tumour, dedifferentiates to form a tumour stem cell, and divides in the process. It comes out already as a tumour stem cell. This type of entosis can also be characteristic of primary tumours during treatment and allows for the rapid regeneration of the cancer stem cell (CSCs) population from differentiated tumour cells. As it is observed after neoadjuvant chemotherapy in breast cancer patients with partial regression compared to patients with stabilisation or progression [66]. In addition, entomammoptosis may serve to dedifferentiate tumour cells of micrometastases in distant organs that have gone dormant. Such dedifferentiation would lead to the formation of CSCs A this would open the way for micrometastases to grow and transform into macrometastases (Fig. 4D). We also identified the leading genes whose expression is associated with overall survival at different localisations (Supplementary Appendix 1, Supplementary Table 1).

Wang et al. included 180 samples from 90 paired tumour (T) and paratumor (P) tissues from hepatocellular carcinoma (HCC) patients. They showed that the mean amount of CIC in tumour tissues (1.78 ± 0.23) was significantly higher than that in paratumor tissues (0.39 ± 0.098) (*p* < 0.001). They also demonstrated that more CICs were detected in HCC tissues with high E-cadherin levels, while no differences in their composition were observed in paratumor tissues. This suggests that homotypic CICs are associated with E-cadherin levels in HCC tissues. Single factor analysis showed that the presence of hoьmoCICs was a strong prognostic factor predicting shorter postoperative survival (mOS: 69 vs. 16.5 months); *p* = 0.0018). This factor was also shown to preferentially predict shorter survival in patients with HCC at early TNM stage (I + II) (*p* = 0.0475), but not at late TNM stage (III + IV) (*p* = 0.0707). And the presence of CICs was significantly associated with low tumour differentiation (*p* = 0.001) [69].

The study by Bauer et al. included 147 female breast cancer (BC) patients with early hormone receptor-positive tumours. TNM stage ranged from T1mic to T2. Entotic events were detected in 61.2% of the examined cases. Interestingly, topographically, the highest proportion of CICs cases were found in the central parts of the tumour (51.6%) and the tumour infiltration zone (41.2%). Patients with CICs in the central tumour zone clearly had a favourable prognosis in terms of local recurrence-free survival (*p* = 0.008) and recurrence-free survival (*p* = 0.027). Multivariate analysis showed that Ki67 (*p* = 0.003) and CIC in central cancer (*p* = 0.018) were independent risk factors for recurrence-free survival. In contrast to this favourable prognostic feature, CIC did not have a similar effect on metastasis-free survival (*p* = 0.498). CIC on the invasive front was not prognostically significant for either recurrence-free survival or metastasis-free survival [70].

In the following work, the entotic indices of tissue microarrays of 50 selected cases of entosis-positive breast cancer were analysed. The total number of entotic indices calculated from scans of the samples was 2.15 times higher in metastasis to lymph nodes than in primary lesions (mean 5.8 vs. 2.7%; *p* < 0.0001). Interestingly, the distribution of entotic structures in the tissue was not homogeneous, and hot spots with increased entosis were observed. They were located in low-differentiated parts of the primary lesion that lacked organotypic structure. Entotic cells were localised preferentially in areas with low to moderate E-cadherin expression. Interestingly, in contrast, a positive correlation between the level of E-cadherin and the number of entotic figures was found in lymph node metastasis. A statistically significant difference in the expression of HER2 receptor and Ki67 depending on the number of entotic figures was observed. Their increased expression was accompanied by an increase in the number of entotic figures in the samples studied; hence, the correlation was positive in both cases [71].

Basbous et al. also investigated the presence of entotic cells in the tissues of HCC patients. Using membrane (pankeratin/β-catenin) and nucleus staining, they detected a small number of entotic cells in tumour samples. They then performed Rnd3 staining on HCC slices and found that entotic cells were present in tumour sections with low Rnd3 expression. Using a group of 10 patient samples from HCC patients, they correlated the number of entotic cells with the characteristics of the patients’ tumours. It was shown that homoCIC counts were significantly higher in tumours with satellite nodules or vascular invasion. All these results suggest a link between entotic events, Rnd3 loss and tumour progression [72].

### The results we have obtained

**Fig. 5:**
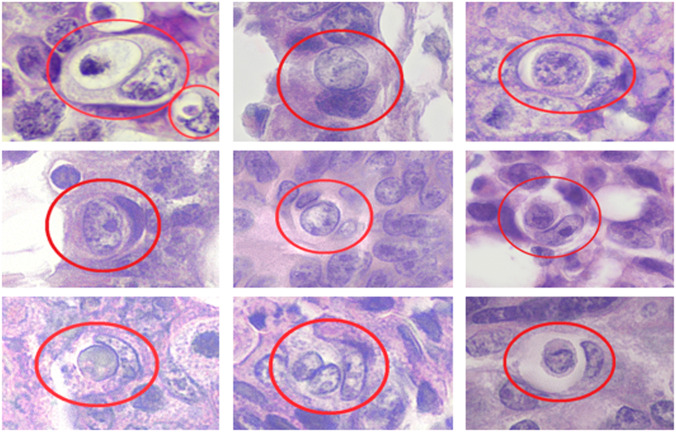
Entosis in samples of colorectal cancer patients. The nuclei of the outer cells are sickle-shaped and pushed to the periphery of the cell. Magnification: 600X. Staining: H/E.

We also conducted a study related to the frequency of entosis in 64 patients with colorectal cancer aged 32–88 years with morphologically verified diagnosis. We analysed histological preparations stained according to the H/E scheme (Fig. 5).

Further, we evaluated the association of entotic event rate with the main clinical and morphological parameters of patients: stage, tumour size, degree of differentiation and Grade. As a result of the analysis, we found that the frequency of entotic events was practically unrelated to clinical and morphological parameters. Statistically significant results were obtained only for tumour size (*p* = 0.03). Consequently, the frequency of CIC structures was higher in T4 tumours, compared to the T2-3 group, respectively.

The overall survival of patients depending on the frequency of entotic events did not show statistically significant results, but indicated a positive trend. We assume that further analysis of the metastatic-free survival may yield a statistically significant correlation. Correlation analysis of 49 CRC patients showed a correlation between the number of stemness gene aplifications (determined using the CytoScanHD Array microarray (Afymetrix, USA)) and the frequency of CIC structures formed. The more entotic structures were detected in the tissue, the more amplifications of stemness genes associated with the ability of tumour cells to metastasise were observed. The Spearman correlation coefficient was 0.47 (*p* = 0.0006).

In connection with the data we have presented, it is important to note that entosis may not only play a role in the molecular mechanism, but also serve as an independent prognostic marker in several cancer localisations, including breast, pancreatic, lung, liver, oesophageal, and head and neck tumours [69, 73–76].

## Conclusion

Due to its poor understanding, entosis has not yet been used in the clinic. However, scientists are actively reflecting on the prospects of its use, with one of the most obvious prospects being the use of entosis as a prognostic marker. In particular, Davies et al. suggest that cellular entotic uptake is an indicator of tumour dissemination capacity. The development of therapeutic strategies based on entotic uptake is still difficult.

The classification we present, if confirmed, can help in the development of entosis-based therapeutic strategies. Stabilising entosis, due to its anti-tumour activity, can be stimulated, thereby enhancing drug therapy. The other types of entosis should certainly be inhibited, but there are nuances in which cases. Transforming entosis should be inhibited during the pre-tumour stages in order to reduce the likelihood of malignisation. Also, transforming entosis should be inhibited during the preoperative treatment of operable forms of tumour and in the 1st and subsequent lines of therapy in disseminated forms, in order to prevent the formation of more malignant tumour clones. Parasitic entosis is unlikely to pose a major threat, as it is a short-term phenomenon, and with modern long-term anti-tumour therapy it will not significantly reduce its efficacy. It seems to us that its inhibition would only be useful in patients with frequently recurrent tumour types, such as laryngeal cancer. Entomammoptosis should be long term inhibited during the completion of adjuvant therapy and long afterwards to prevent the dedifferentiation of micro-metastatic tumour cells emerging from the dormant state.

Overall, entosis is a very interesting phenomenon that requires extensive research both clinically and experimentally, and these studies may provide insights into the mechanisms of tumour progression, resistance formation and will contribute to the development of new therapeutic strategies.

### Supplementary information

Appendix 1. Table 1
