# Supplementary material for: Mechanisms and significance of entosis for tumour growth and progression
Source: Cell Death Discov. 2024 Mar 1;10:109. doi: 10.1038/s41420-024-01877-9 (PMC10907354; doi:10.1038/s41420-024-01877-9)
Supplement: Supplementary file 1 — Appendix 1. Table 1 [file 41420_2024_1877_MOESM1_ESM.docx]

Application 1. Table 1. Expression of genes involved in the realisation of entosis, association with overall survival rate.

| Localisation | Basic genes | | | | | | |
| --- | --- | --- | --- | --- | --- | --- | --- |
|  | *RAC1* | *MYC* | *CDH1* | *MAP1LC3A* | *ROCK1* | *CDC42* | *RhoA* |
| Bladder Carcinoma | P value: 0.0121 HR: 1.49 (1.09-2.05) | P value: 5.8e-5 HR: 1.81 (1.35-2.43) |  |  |  |  |  |
| Breast cancer | P value: 0.0047 HR: 1.59 (1.15-2.21) | P value: 0.0303 HR: 0.7 (0.51-0.97) | P value: 0.0437 HR: 1.4 (1.01-1.94) | P value: 0.004 HR: 0.63 (0.45-0.86) |  |  |  |
| Esophageal Squamous Cell Carcinoma |  |  |  | P value: 0.0207 HR: 4.74 (1.11-20.22) |  |  |  |
| Head-neck squamous cell carcinoma |  | P value: 0.0034 HR: 1.53 (1.15-2.03) |  | P value: 0.0107 HR: 0.69 (0.52-0.92) |  |  |  |
| Cervical squamous cell carcinoma |  | P value: 0.004 HR: 2.02 (1.24-3.29) |  |  | P value: 0.0391 HR: 1.84 (1.02-3.3) |  |  |
| Esophageal Squamous Cell Carcinoma |  |  | P value: 0.0142 HR: 2.58 (1.18-5.67) |  | P value: 0.0158 HR: 0.38 (0.17-0.86)" |  |  |
| Kidney renal clear cell carcinoma | P value: 0.0002 HR: 1.79 (1.31-2.44) | P value: 0.0004 HR: 2.81 (1.55-5.12) | P value: 3.1e-5 HR: 0.54 (0.4-0.72) | P value: 0.0513 HR: 1.34  (1-1.81) | P value: 2.4e-5 HR: 0.5 (0.36 -0.7) | P value: 8.6e-8 HR: 0.45 (0.33-0,61) | P value: 1.8e-6 HR: 0.48 (0.36-0.66) |
| Kidney renal papillary cell carcinoma |  |  | P value: 0.007 HR: 2.22 (1.22-4.02) | P value: 0.0008 HR: 0.37 (0.21-0.68) |  |  | P value: 0.0006 HR: 0.37 (0.2-0.67) |
| Liver hepatocellular carcinoma | P value: 2.3e-5 HR: 2.52 (1.62-3.92) | P value: 0.0579 HR: 1.43 (0.99-2.08) | P value: 0.005 HR: 0.61 (0.43-0.86) |  |  | P value: 5.3e-5 HR: 2.05 (1.44-2.94) | P value: 0.0006 HR: 1.91 (1.29-2.56) |
| Lung adenocarcinoma | P value: 6.5e-6 HR: 1.94 (1.45-2.6) | P value: 0.0349 HR: 1.38 (1.02-1.86) |  | P value: 0.0332 HR: 0.68 (0.47-0.97) |  |  |  |
| Lung squamous cell carcinoma |  |  |  |  |  |  | P value: 0.0384 HR: 1.34 (1.01-1.77) |
| Ovarian cancer |  | P value: 0.0008 HR: 1.61 (1.22-2.13) |  |  | P value: 0.0077 HR: 0.68 (0.51-0.9) | P value:0.0188 HR: 0.69 (0.5-0.94) | P value: 0.0104 HR: 0.7 (0.53-0.92) |
| Pancreatic ductal adenocarcinoma | P value: 4.6e-5 HR: 2.75 (1.66-4.56) | P value: 0.0061 HR: 1.85 (1.18-2.89) | P value: 0.0119 HR: 1.86 (1.14-3.03) | P value: 2.8e-5 HR: 0.39 (0.25-0.62) | P value: 0.001 HR: 2.14 (1.35-3.39) | P value: 0.0563 HR: 1.5 (0.99-2.27) | P value: 0.0287 HR: 1.58 (1.05-2.39) |
| Pheochromocytoma and Paraganglioma" | P value: 0.0213 HR: 0.17 (0.03-0.94) |  |  | P value: 0.0026 HR: 0.07 (0.01-0.64) |  | P value: 0.0057 HR: 0 (0-lnf) |  |
| Rectum adenocarcinoma |  | P value: 0.0189 HR: 0.41 (0.19-0.88) | P value: 0.0006 HR: 0.12 (0.03-0.51) |  |  |  | P value: 0.0029 HR: 0.33 (0.15-0.71) |
| Sarcoma | P value: 0.0154 HR: 1.82 (1.11-2.98) | P value: 0.0011 HR: 1.91 (1.29-2.85) | P value: 0.0415 HR: 0.66 (0.44-0.99) | P value: 0.0071 HR: 0.48 (0.28-0.83) |  | P value: 0.0037 HR: 1.93 (1.23-3.03) |  |
| Thymoma |  | P value: 0.0066 HR: 0.17 (0.04-0.71) |  | P value: 0.0012 HR: 7.25 (1.79-29.35) | P value: 0.0042 HR: 0.16 (0.04-0.67) | P value: 0.0592 HR: 0.24 (0.05-1.19) | P value: 0.0009 HR: 0.11 (0.02-0.53) |
| Thyroid carcinoma | P value: 0.041 HR: 0.36 (0.13-1) |  |  |  |  | P value: 0.0543 HR: 2.72 (0.947.84) |  |
| Uterine corpus endometrial carcinoma | P value: 0.0181  HR: 0.6 (0.39-0.92) | P value: 0.0028 HR: 1.89 (1.23-2.88) |  | P value: 0.0201 HR: 0.59 (0.38-0.93) |  | P value:0.0511 HR: 0.66 (0.43-1.01) |  |
| Stomach adenocarcinoma |  |  |  |  | P value: 0.0201 HR: 1.48 (1.06-2.06) |  |  |
| Testicular Germ Cell Tumor | P value: 0.0211  HR: 0 (0- lnf) |  |  |  | P value:0.0547 HR: 73533984.01 (0-lfn) |  |  |
